# Myocarditis, Disseminated Infection, and Early Viral Persistence Following Experimental Coxsackievirus B Infection of Cynomolgus Monkeys

**DOI:** 10.1371/journal.pone.0074569

**Published:** 2013-09-09

**Authors:** Cheryl E. Cammock, Nancy J. Halnon, Jill Skoczylas, James Blanchard, Rudolf Bohm, Christopher J. Miller, Chi Lai, Paul A. Krogstad

**Affiliations:** 1 Department of Pediatrics, Wake Forest University School of Medicine, Winston-Salem, North Carolina, United States of America; 2 Department of Pediatrics, David Geffen School of Medicine at University of California, Los Angeles, California, United States of America; 3 Department of Pathology and Laboratory Medicine, David Geffen School of Medicine at University of California, Los Angeles, California, United States of America; 4 Department of Molecular and Medical Pharmacology, David Geffen School of Medicine at University of California, Los Angeles, California, United States of America; 5 Department of Veterinary Medicine, Tulane University National Primate Research Center, Covington, Louisiana, United States of America; 6 California National Primate Research Center and Center for Comparative Medicine, University of California, Davis, California, United States of America; University of California, San Francisco, United States of America

## Abstract

Coxsackievirus B (CVB) infection is a common cause of acute viral myocarditis. The clinical presentation of myocarditis caused by this enterovirus is highly variable, ranging from mildly symptoms to complete hemodynamic collapse. These variations in initial symptoms and in the immediate and long term outcomes of this disease have impeded development of effective treatment strategies. Nine cynomolgus monkeys were inoculated with myocarditic strains of CVB. Virological studies performed up to 28 days post-inoculation demonstrated the development of neutralizing antibody in all animals, and the presence of CVB in plasma. High dose intravenous inoculation (n = 2) resulted in severe disseminated disease, while low dose intravenous (n = 6) or oral infection (1 animal) resulted in clinically unapparent infection. Transient, minor, echocardiographic abnormalities were noted in several animals, but no animals displayed signs of significant acute cardiac failure. Although viremia rapidly resolved, signs of myocardial inflammation and injury were observed in all animals at the time of necropsy, and CVB was detected in postmortem myocardial specimens up to 28 days PI. This non-human primate system replicates many features of illness in acute coxsackievirus myocarditis and demonstrates that myocardial involvement may be common in enteroviral infection; it may provide a model system for testing of treatment strategies for enteroviral infections and acute coxsackievirus myocarditis.

## Introduction

Viral infections are the most common etiology of acute myocarditis. Parvovirus B19, human herpes virus 6, adenoviruses, and the non-polio enteroviruses have been most frequently implicated in recent studies [1]. Among the enteroviruses, the group B coxsackieviruses (CVB) have historically warranted great attention due to the age-dependent differences in the outcome of acute infection. In the newborn period, these viruses often produce life-threatening disease including meningoencephalitis, hepatitis, sepsis and myocarditis [2], [3], [4]. Although the circulation of the many serologic types of enteroviruses shows year to year variation [2], a 2007 outbreak of cases of CVB type 1 (CVB1) in newborns and other recent reports [5], [6], [7], [8] demonstrate the ongoing threat posed to newborns by myocarditic coxsackieviruses. In contrast, older children and adults with enteroviral myocarditis typically present with less severe initial disease and typically have better long-term outcomes [9].

Mechanistic studies in inbred strain specific murine models have suggested the possibility of progression from acute viral myocarditis to chronic dilated cardiomyopathy after infection with CVB, but confirmatory human data or demonstration of chronic viral infection or latency in genetically heterogeneous animal models are lacking [10], [11], [12]. Other animal models of myocarditis exist, including the induction of autoimmune myocarditis in Lewis rats by injection of myosin, and acute infection of pigs by encephalomyocarditis virus (EMCV) [13], [14]. However, EMCV is only rarely a pathogen in humans, and the Lewis rat system does not model the impact of viral replication in the myocardium and other organs. Consequently, these systems are far from ideal models of the acute pathophysiology and sequelae of enterovirus infection in humans.

By contrast, non-human primates have many immunological and physiological similarities with humans that might facilitate their use in enterovirus research, including the structure and function of immunoglobulins [15], organization of major histocompatibility antigen families [16], and cardiac physiology [17]. CVB infection of non-human primates has been described in several reports. In 1983, Hoshino et al described experimental infection of 11 cynomolgus monkeys with CVB and detected electrocardiographic changes and diffuse inflammatory infiltrates in the myocardium, particularly in the right ventricle [18]. In addition, there have been case reports of non-human primates with naturally acquired infection of coxsackievirus and disease similar to those in humans [19], [20]. However, these reports preceded the development of current virological and immunological methods.

Natural history studies lack an accurate estimate of the prevalence and severity of disease after infection with (versus ‘exposure to’) enterovirus. In this report, we describe the prevalence of infection and myocarditis and immunological, virological, and pathologic events seen following infection of cynomolgus monkeys with two different strains of CVB, including a strain well known to induce myocarditis in mice and a recently identified strain from a case of fatal neonatal myocarditis [6]. These studies delineate a model for examination of virus-induced pathology in non-human primates infected with cardiotropic viruses. Such a model will be useful for preclinical and mechanistic testing of potential treatment strategies.

## Methods

### Animals

Nine female cynomolgus monkeys (Macaca fascicularis) lacking neutralizing antibody to coxsackievirus B3 (CVB3) were randomly selected for use in this study. The animals ranged in age from 7.3 to 9.5 years and had body weights of 2.0 to 3.3 kg. All animals were housed at the Tulane National Primate Research Center (TNPRC). The TNPRC is an Association for Assessment and Accreditation of Laboratory Animal Care accredited facility (AAALAC #000594). The OLAW animal welfare assurance number for TNPRC is A4499-01 and the USDA registration number is 72-R-0002. The TNPRC animal care program follows the laws and guidelines of the US Animal Welfare Act and the NIH Guide for the Care and Use of Laboratory Animals. The study presented in this manuscript was approved by the TNPRC IACUC and by the Institutional Animal Care and Use Committee of the University of California, Los Angeles.

Animal care at TNPRC is provided by a faculty of 8 veterinarians (7 ACLAM Diplomates), and 110 animal care technicians, veterinary technicians, and enrichment staff. Animals on this study were pair housed and received standard enrichment which includes manipulable items in cage (durable and destructable objects), perches or swings, various food supplements (fruit, vegetables, primate treats), foraging or task- oriented feeding methods, and human interaction with caretakers and research staff. Monkeys are fed commercially produced chow twice a day. Post IACUC approval monitoring including procedure evaluation, assessment of technical skill and adherence to SOPs is assessed by veterinary faculty and staff. Veterinarians are available 24 hours a day to provide emergency care. The TNPRC Division of Veterinary Medicine has established procedures to minimize pain and distress through several means. The use of preemptive and post procedural analgesia is required for procedures that would likely cause more than momentary pain or distress in humans undergoing the same procedure. Any deviation from the administration of analgesics according to this policy requires adequate scientific justification from the investigator and approval by the IACUC. Tulane has a written endpoint policy to minimize potential pain and distress experienced by animals. The policy addresses limits on weight loss, appetite, tumor size, response to medical intervention, activity, and a number of other clinical signs relevant to laboratory animal species. If an animal becomes ill and/or meets the criteria for the IACUC approved endpoint policy, it is euthanized using methods consistent with recommendations of the American Veterinary Medical Association (AVMA) Panel on Euthanasia. Briefly, animals are anesthetized with ketamine hydrochloride (10 mg/kg) and given an overdose of sodium pentobarbital.

### Virus Preparations

Infectious stocks of the myocarditic H3 strain of CVB3 were produced by co-transfection of Hela cells with a plasmid clone of the genome of this strain (pH3), along with a plasmid encoding T7 RNA polymerase (pAR3126), and titered on Hela-RW cells as described by others [21], [22], [23]. A stock of CVB3-MCH (a myocarditic isolate recovered from a case of fatal neonatal myocarditis at Mattel Children’s Hospital (MCH) at UCLA in 2005 [6]) was prepared and titered using HeLa-RW cells. Animals were infected by injection via the saphenous vein (8 animals) or by enteral inoculation via an 8 French orogastric tube under ketamine HCl anesthesia (1 animal).

### Clinical evaluation

Animals were anesthetized with ketamine HCl prior to inoculation with CVB3, and at each subsequent study point. At each time point, transthoracic echocardiography was performed using the LOGIQ Book XP (GE Healthcare, USA) with an 8 mhz transducer. Wall motion was qualitatively examined by 2-dimensional images in the parasternal long and short axis (below the mitral valve) views and ejection fraction and fractional shortening were measured by M mode in the parasternal short axis view at the level of the mitral valve leaflets. Additional views were examined when images were of sufficient quality. While anesthetized, venous blood samples were drawn for complete blood count, liver function tests, creatine phosphokinase (CPK), blood glucose, assessment of serum neutralization antibodies, and detection of CVB3 by culture and RT-PCR. Following infection, the animals were monitored daily for activity level and appetite. Body temperature was measured on days when the animals were anesthetized. Point of care measurement of troponin I, B-type natriuretic peptide (BNP) and CPK-MB fraction were performed on plasma and serum using the Biosite Triage BNP Test (Biosite Inc., San Diego, CA) (Limits of detection for these analytes were as follows: TNI, 0.05 ng/ml; BNP: 5 pg/ml; and CPK-MB 1 ng/ml). Plasma specimens TNI levels were later reexamined using a monkey TNI ELISA assay kit (Life Diagnostics Inc., West Chester, PA) with a sensitivity level of 0.15 ng/ml. All measurements were repeated 3, 7, 14 and, in some cases, day 28 post-inoculation.

### Detection of CVB Neutralizing antibody

CVB3-specific antibody levels in the serum of the animals were determined by a microneutralization assay. Fourfold dilutions of heat-inactivated serum from each time point were prepared ranging from 1∶10 to 1∶163,840 and added to an equal volume of virus containing 150 plaque forming units (pfu) of CVB (H3) was added to the wells of a 96 well plate. After incubation at 37°C for 2 hours, Hela-RW cells were added to each well (50,000 cells in 200 µl of medium). After 48 hours, the wells were examined, and the neutralizing antibody titer was designated as the highest dilution that prevented the development of cytopathic effect (CPE).

### Quantitative RT- PCR detection of CVB RNA

RT-PCR was performed for CVB3 RNA in plasma collected at day 3, 7 and 14 post-inoculation (PI) and cardiac tissue samples obtained at the time of necropsy in all infected animals. Viral RNA was purified from 140 to 280 microliters of plasma using QiAmp Viral RNA mini kit (Qiagen, USA) as specified the manufacturer. Fresh frozen cardiac tissue was homogenized in TRIzol reagent (Invitrogen Life Technologies Inc., USA), and total RNA was extracted. The concentration of total RNA was determined by spectrophotometery. cDNAs were generated by reverse transcriptase reaction using random hexamer primers, and CVB sequences were amplified and quantified by Real Time RT-PCR as described by others [24] using serially diluted *in vitro* transcribed CVB3 RNA as quantitative standards. The limits of detection were approximately 17,000 copies of CVB3 RNA/ml plasma.

### Detection of infectious virus in cynomolgus monkey plasma and cardiac tissue

Frozen plasma specimens from each time point were thawed, vortexed, and added to HeLa-RW cell culture. Frozen fragments of cardiac tissue were thawed, resuspended in tissue culture medium, and mechanically disrupted using a Mini-Beadbeater-1 (Biospec, Bartlesville, OK). Clarified supernatants were added to HeLa-RW cells, incubated for 24 to 72 h at 37°C and observed for characteristic CPE. All cultures were also blindly passaged by scraping the cells in each well into the overlying medium. After three freeze-thaw cycles, the lysates were clarified by centrifugation and added to new cell cultures. After 48 hours incubation at 37°C, the cultures were again examined to detect the presence of CPE.

### Plasma Cytokine Evaluation

The concentration of plasma cytokines in frozen plasma samples was performed using a Luminex multiplex cytokine assay (Millipore) to detect cynomolgus IL1-beta, IL-1_ra_, TNF-alpha, IFN-gamma, IL-2, IL-4, IL-5, IL-6, IL-8, IL-10, IL-12/23 (p40), IL-13, IL-15, IL-17, IL-18, the chemokines MCP-1, MIP-1 alpha, MIP-1 beta, as well as VEGF, G-CSF,GM-CSF, CD40L, and TGF- alpha. The limit of detection for each analyte was 1 to 3 pg/ml.

### Pathologic evaluation: histology and immunohistochemistry

Animals were sacrificed at the times indicated. Necropsy was performed on all animals used in the study. Complete histological examination of all major organs was performed. For immunohistochemical evaluation paraffin-embedded sections were stained using the Dako Autostainer (Dako Inc., Carpenteria CA). The primary antibodies used included polyclonal anti-CD3 rabbit serum (Dako Inc. Carpinteria CA),and mouse anti-human anti- CD4 (Vector, Burlingame, CA) CD8 (Leica Microsystems, Bannockburn, IL), CD68 (Dako, Inc. Carpinteria CA) and CD20 (BD Biosciences, San Jose CA). For all primary antibodies, slides were subjected to an antigen retrieval step consisting of incubation in AR10 (Biogenex Inc, San Ramon, CA) for 2 min at 123°C followed by cooling to 90°C before rinsing in running water and a final buffer rinse. Primary antibodies were replaced by normal rabbit (Zymed Inc., South San Francisco CA) or mouse IgG (Dako, Inc., Carpinteria CA) and included with each staining series as the negative control. Binding of the mouse antibody was detected using EnVision anti-mouse polymer (Dako, Inc. Carpinteria CA) or biotinylated sheep anti-rabbit IgG (Vector Lab Inc.), Streptavidin-HRP (Invitrogen Inc. Grand Island NY) and DAB (Dako Inc. Carpinteria CA). All slides were counterstained with hematoxylin. Control experiments gave appropriate results with minimal non-specific staining.

## Results

### Disseminated disease after high dose intravenous inoculation

We initially infected two animals by intravenous injection of 10^7^ pfu of H3, a strain of CVB3 known to be myocarditic in mice [22], [23]. Inoculation with this quantity of another strain of CVB was previously shown to produce infection in 70% of nonhuman primates (both baboons and cynomolgus monkeys) [18], [25]. These two animals developed signs of illness by 7 days PI, including anorexia and decreased activity. One animal (FP49) was found to be hypothermic, bradycardic, and moribund on day 11, and was euthanized. The second animal (FP42) was euthanized at day 14 to look for evidence of virus induced pathology.

**Figure 1:**
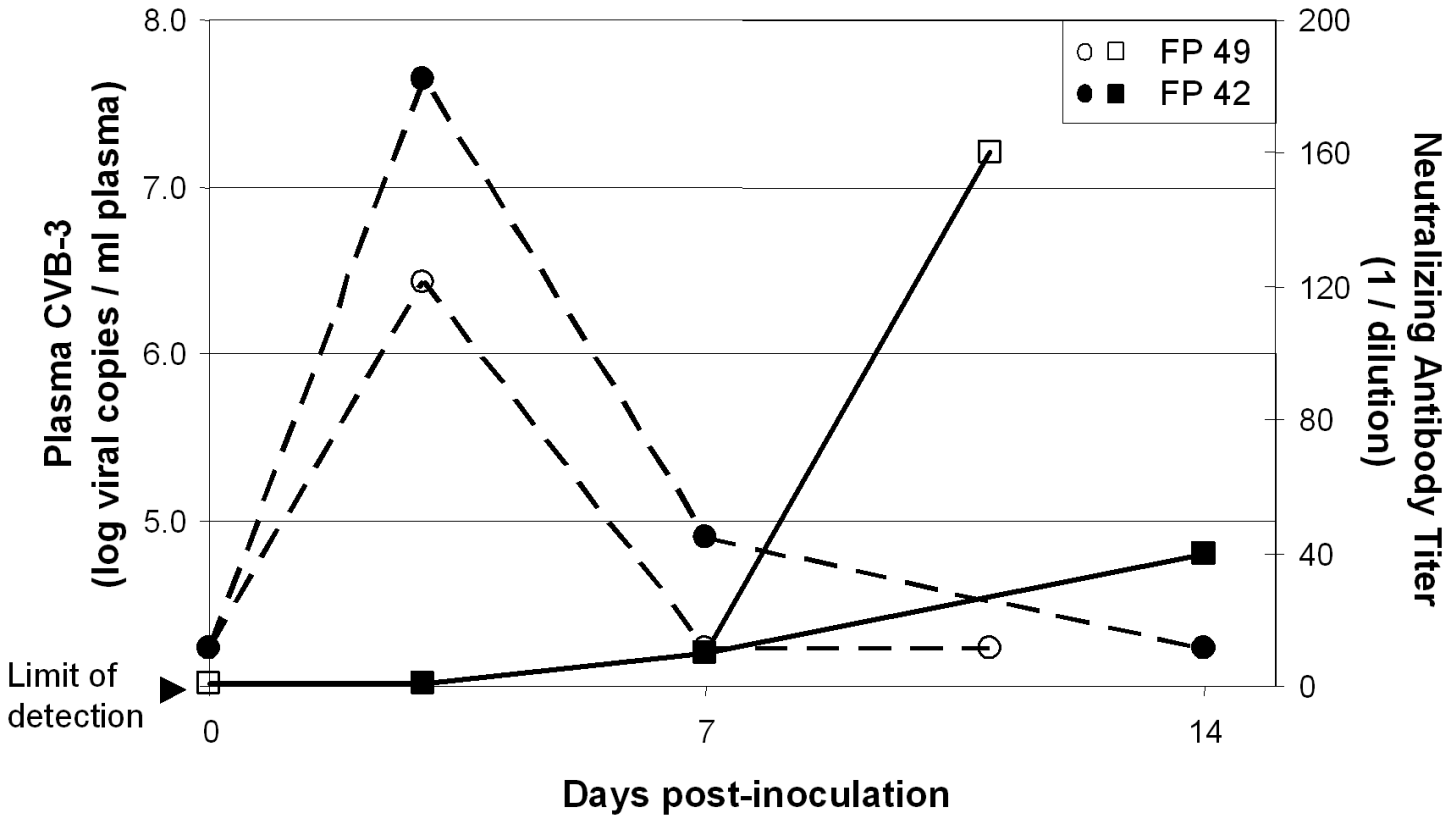
Coxsackievirus viral load (plasma viral RNA copies/ml) (dashed lines) and neutralizing antibody titers (reciprocal of dilution) (solid lines) after intravenous infection of two cynomolgus monkeys: FP42 (closed figures) and FP49 (open figures).

**Figure 2:**
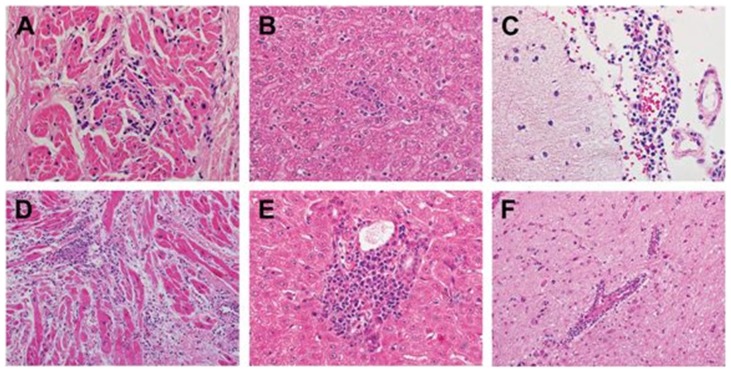
Postmortem histopathologic findings (hematoxylin-eosin (H&E) stain) of two cynomolgus monkeys injected with 10^7^ pfu of CVB3-H3. Myocardium from both FP42 (A (200X)) and FP49 (D (200X)) showed evidence of viral myocarditis, consisting of focal predominantly lymphocytic mononuclear inflammatory infiltrates with myocyte injury. In addition, representative sections of the liver from both animals exhibited changes of mild viral hepatitis including focal mononuclear inflammatory infiltrates within the hepatic lobules (B (FP42)) and portal tracts with associated interface hepatitis (E (FP49)). The central nervous system exhibited perivascular lymphocytic infiltrates within the leptomeninges (C (FP42)) and intracerebral blood vessels (F (FP49)) with microglial proliferation and nodules (not shown). These changes are indicative of viral meningitis and encephalitis (meningoencephalitis).

Both animals were viremic on the third day PI, as demonstrated by both RT-PCR and culture (Figure 1, Table 1). They developed neutralizing antibody by day 7, and plasma viral cultures were negative on day 7 and the day of euthanasia. Necropsy revealed myocarditis, as expected, but there was also histological evidence of meningitis, encephalitis, pancreatitis, and hepatitis in both animals (Figure 2). Thus, in these animals, CVB3 infection produced disseminated illness, similar to that seen following CVB infection of newborn infants. Although there was clear histological evidence of myocarditis at necropsy, cardiomegaly was not noted and no significant echocardiographic or electrocardiographic abnormalities were found, apart from minor transient wall motion abnormalities (localized segmental hypokinesis) noted in FP49 on the third day following infection. Ejection fraction and shortening fraction measurements remained unchanged from baseline. Neither animal had detectable plasma concentrations of TNI or BNP at baseline or the day of euthanasia.

**Table 1:** Summary of Animals and Virologic Studies.

Animal	Virus	Inoculum(pfu)	Route	Euthanized(study day)	CVB3 RNA(copies/ml plasma)^1^	PlasmaCulture	RT-PCRCardiac Tissue^2^
FP42	CVB-H3	10^7^	IV	14	4.5×10^7^	+	-^3^
FP49	CVB-H3	10^7^	IV	11	2.7×10^6^	+	LV,RV,
FP41	CVB-H3	10^6^	IV	14	-	-	LV,RV
FP44	CVB-H3	10^6^	IV	14	1.0×10^6^	+	RV
FP46	CVB-H3	10^6^	IV	28	-	-	RA,RV
FP43	CVB-H3	10^7^	Enteral^4^	14	8.1×10^5^	+	LA
FP47	CVB-MCH	10^6^	IV	28	5.5×10^4^	-	-
FN16	CVB-MCH	10^6^	IV	28	3.8×10^6^	+	-
FN20	CVB-MCH	10^6^	IV	28	4.0×10^5^	+	-

1. Limit of detection approximately 10,000 copies/ml. “**-**“  =  not detected.

2. Viral RNA detected in tissue from chamber designated. LA =  left atrium, RA  = right atrium, LV  =  left ventricle, RV  =  right ventricle.

3. Only RV, LV, and RA tissue available from this animal.

4. See text for description.

### Myocardial injury and subclinical disease following low dose intravenous CVB3 infection

**Figure 3:**
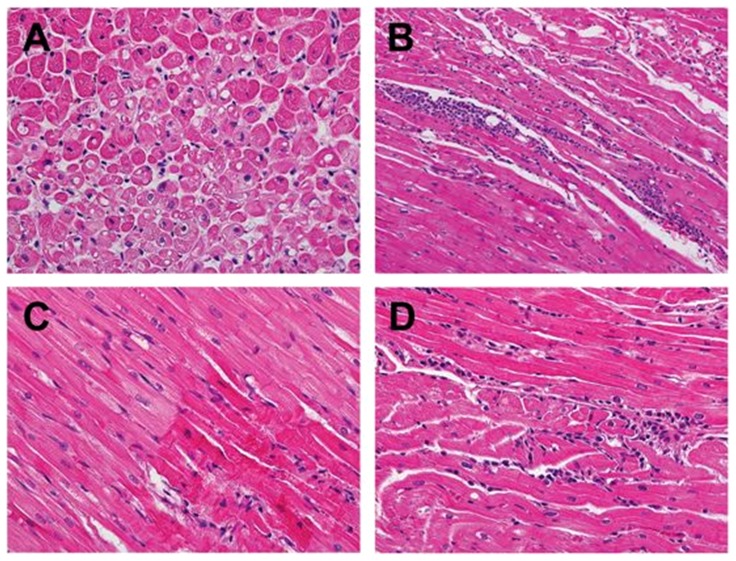
Histopathological changes in the myocardium of animals infected with CVB-H3 (FP41, FP44, and FP46) and CVB3-MCH (FP47). FP41 (A) exhibiting prominent cytoplasmic vacuolization indicative of myocytolysis (400X; H&E stain). FP44 (B) revealing focal, predominantly lymphocytic, mononuclear inflammatory infiltrate with associated myocyte injury consistent with myocarditis (400X; H&E stain). FP46 (C) showing hypereosinophilic, hypercontracted myofibrils indicative of contraction band necrosis of cardiac myocytes (right lower corner) (400X; H&E stain). FP47 (D) demonstrating focal mononuclear inflammatory infiltrate without associated myocardial injury (400X; H&E stain).

In view of the severe illness observed in the initial pair of animals, the viral inoculum was reduced ten-fold to 10^6^ pfu, and three monkeys (FP41, FP44, and FP46) were infected by intravenous injection of CVB3-H3 and euthanized either 14 days (FP41, FP44) or 28 days later (FP46). At this dose, viremia was demonstrated in only one of these animals, and none developed signs of illness after inoculation. Nonetheless, all three had evidence of myocardial injury at the time of necropsy (Figure 3, Table 1). In FP41 there were foci of vasculitis and myocarditis as well as localized hypertrophy and myocytes with enlarged nuclei. In FP44 myocardial findings included hypertrophic myocytes, areas of inflammation without necrosis, and foci of lymphocytic myocarditis (inflammatory infiltrates and injury to myocytes). FP46 had similar evidence of inflammation, hypertrophy, and ischemia at the time of necropsy (28 days PI). CVB RNA was detected in the myocardium of FP44 and FP46 at 14 and 28 days after infection respectively. TNI and BNP were not detected in the plasma of either of these animals at any time point, but both had echocardiographic evidence of minor wall motion abnormalities only on day 3 PI. Ejection fraction and shortening fraction remained unchanged from baseline.

To determine if other myocarditic strains would produce similar pathology, three additional animals (FN16, FN20, FP47) were injected with 10^6^ pfu of CVB3-MCH, a strain recovered in 2005 from a newborn infant that died of myocarditis [6]. CVB3 RNA was detected in plasma from all three on the third day after inoculation, and two had positive plasma viral cultures as well (Table 1). The animals were euthanized four weeks after inoculation. Histological abnormalities found in cardiac tissue at necropsy included focal cellular infiltrates in the myocardium of FP47 (Figure 3, panel D), an area of ischemic change in FN 16 and myocyte hypertrophy in FN 20 (not shown). CVB genomes were not detectable by PCR in these animals and all other organs examined were free of significant pathologic lesions at the time of necropsy (day 28 PI). However, CVB-MCH was detected by viral culture of tissue from the left ventricle of FP47 when necropsy was performed on day 28 post-infection.

**Figure 4:**
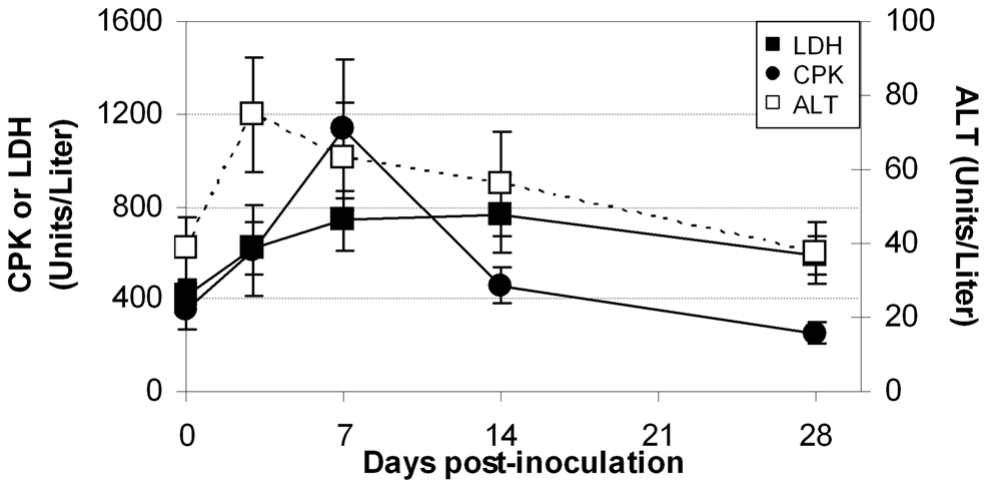
Biomarkers measured in animals inoculated with low dose (10^6^ pfu) CVB-H3 or CVB-MCH. Serum, unfractionated LDH (▪), total CPK (•), and ALT (□) are represented as mean +/− SEM for all 6 animals at each timepoint.

CPK, lactate dehydrogenase (LDH), and alanine aminotransferase (ALT) were measured in all six animals infected with either low dose CVB3-H3 or CVB3-MCH (Figure 4). The serum ALT increased in all but one animal during the period of follow-up; a two fold elevation was typically seen. All animals developed an increase in both serum CPK and LDH, reaching their maximum observed values by day 7 and 14, respectively. Isozyme fractionation of LDH was not performed. FP41 and FP44 both had histologic evidence of hepatitis and FP41 had minimal focal pancreatitis at necropsy (not shown). FP46 had normal histologic findings in the liver, similar to those animals infected with high dose virus and examined on days 11 or 14 PI. In contrast, none of the animals euthanized on day 28 had histologic evidence of hepatitis after infection with either virus.

### CVB3 infection after enteral inoculation

In addition to the eight animals infected by intravenous injection, one animal (FP43) was inoculated with 10^7^ pfu of CVB3-H3 by placing half of the virus suspension in the oropharynx, and half in the stomach via a feeding tube. Viremia was also demonstrated in this animal on day 3, and CVB3 RNA was detected in the left atrium at 14 days PI when the animal was euthanized (Table 1). Histopathologic findings included scattered areas of myocytolysis and contraction band necrosis in the heart as well as evidence of multifocal lymphocytic hepatitis (not shown).

### Immunohistochemistry

**Figure 5:**
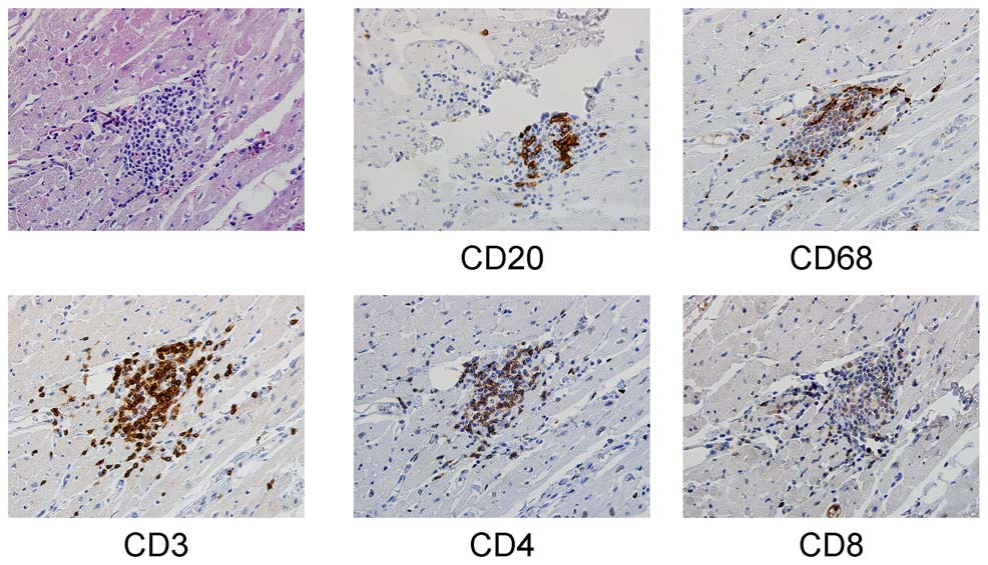
Immunohistochemistry of myocardial tissue. Upper left panel: H&E stain. Antibody specificity used for immunoperoxidase staining is listed below remaining panels. (All sections at 400X)

Myocardial specimens containing inflammatory foci were subjected to immunohistochemical analysis to determine characteristics of cellular infiltrates. Examination of tissue from the right atrium of one animal infected with high dose virus (FP42) demonstrated a predominantly mononuclear infiltrate containing CD3 cells with CD4 and CD8+ T cells approximately equally represented. Monocytes were present in the infiltrate and in areas surrounding it. B cells were nearly absent from the infiltrate and surrounding myocardium (Figure 5). Inflammatory infiltrates from other animals (FP49, FP41, FP44, FP46) infected with both high and low dose of virus and examined at 14 or 28 days revealed similar results: infiltrates consisting of a combination of CD4+ and CD8+ T cells in equal proportions, many macrophages within the infiltrate and scattered around injured myocardium, and a near complete absence of B cells.

### Immunological and Virological Analysis

As noted above, viremia was demonstrated by culture of plasma collected on the third day after inoculation in 6 of 9 animals; cultures of plasma on all subsequent time points were negative. RT-PCR demonstrated the presence of CVB3 RNA in plasma in 7 of 9 animals on Day 3 PI. All 9 animals developed neutralizing antibody by day 7 with titers ranging from 1∶10 to 1∶160, (data not shown) and none had evidence of viremia by either RT-PCR or culture at day 7 or at subsequent timepoints, except FP42 (Figure 1). This animal had the highest level of viremia at day 3 and viral RNA was again detected in plasma on day 7.

**Figure 6:**
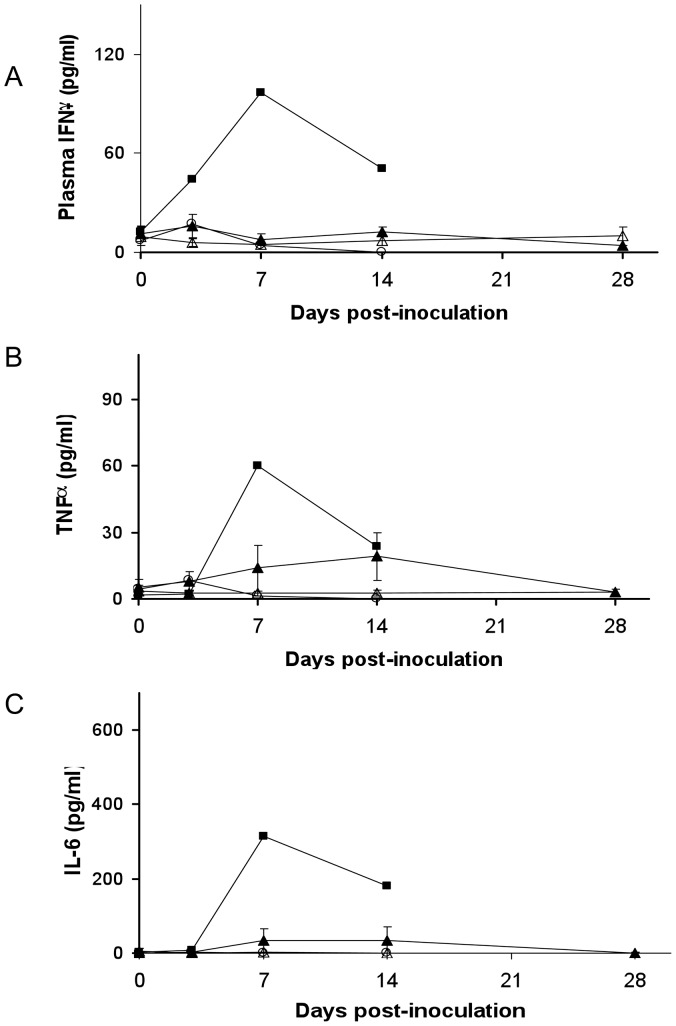
Plasma cytokine concentrations following intravenous inoculation with CVB3. Concentrations of single cytokines IFN-g (panel A), TNF-a (Panel B), and IL-6 (panel C) are demonstrated. Mean cytokine concentrations for the two animals receiving high dose (10^7^ pfu) CVB-H3 by i.v. (▪) or the single animal infected by enteral infection (**○**) are shown. Mean cytokine concentration +/− SEM for animals receiving low dose (10^6^ pfu) CVB-H3 (▴) or CVB-MCH (Δ) by i.v. are shown

Multiplex cytokine assays were used to examine changes in the plasma concentration of proinflammatory and immunomodulatory cytokines and chemokines and their relationship to plasma viremia. Most of the 23 analytes were undetectable or present at low, constant concentrations in plasma throughout the period of observation. Striking changes in the plasma concentration of the proinflammatory cytokines IL-6, TNFalpha, and interferon gamma were identified in the animals infected with CVB3-H3 by high dose intravenous inoculation (FP42 and FP49). Peak plasma cytokine concentrations were present on day 7 (Figure 6). Only one of the animals given low dose or oral inoculation (FP41) exhibited significant changes in any of the plasma cytokines evaluated, despite the near uniform presence at necropsy of histological changes indicative of myocardial injury as well as hepatic injury in those animals examined by day 14.

## Discussion

In this report we describe the clinical and histopathologic characteristics of acute infection of non-human primates with myocarditic strains of CVB. High dose intravenous inoculation of cynomolgus monkeys resulted in severe, disseminated disease in which a brief period of viremia was followed by encephalitis, myocarditis, and other foci of infection, mimicking the pattern of disseminated disease seen in neonatal infection [2]. By contrast, oral or low dose intravenous inoculation of either strain of CVB3 studied resulted in subclinical illness that included evidence of mild hepatitis and a variety of patterns of myocardial injury. No visible myocardial scars or evidence of either ventricular enlargement or myocardial thickening was observed during necropsy. While inflammatory lesions are commonly encountered in the subendocardial or supepicardial tissue in cynomolgous monkeys [26], inflammation or definite myocarditis (as defined by the Dallas Criteria [27]) were present in most animals (FP42, FP49, FP41, FP44, FP46, FP47). Other myocardial abnormalities were also noted, including myocytolysis, contraction band necrosis, and hypertrophy of either myocytes or myocyte nuclei. Inflammatory infiltrates consisted of CD4+ and CD8+ T cells with many macrophages and were similar in tissues from animals examined at 2 or 4 weeks after infection. These processes appeared to affect only a small area of the myocardium. Although we did not examine cytokine levels in the myocardium, this may explain why plasma cytokine levels only changed substantially in the two animals infected with high-dose CVB3.

Our experience provides evidence of CVB persistence in the myocardium. Inoculation of cynomolgus monkeys with 10^6^ pfu of either of the myocarditic strains of CVB3 was followed by rapid resolution of viremia (demonstrated by RT-PCR detection of viral RNA) and subclinical infection. Nonetheless, clearance of CVB from the primate myocardium was protracted, with CVB RNA detected in the myocardium of 5 of 9 animals at 14−28 days, and recovery of virus by culture from one animal (FP47) at 28 days PI. It may be argued that detection of only positive strand RNA from enterovirus is not incontrovertible evidence of persistent infection, but the characteristics of replication of these viruses make detection of negative strand difficult: The ratio of positive to negative strand RNA during is 100 fold per replication cycle making it exceedingly difficult to assure detection of negative strand without contamination by positive strand [28]. Furthermore, one animal had a positive culture from myocardial tissue, providing direct evidence of persistent viral infection.

Our understanding of the natural history of viral myocarditis in humans is incomplete. In the short term, patients with fulminant myocarditis may present with dramatic clinical symptoms, but generally have a good long-term prognosis if supportive therapies allow survival in the face of hemodynamic instability [29], [30]. Based on murine models and case reports, it has been proposed that dilated cardiomyopathy may begin with mild, perhaps unrecognized, enterovirus infections followed by persistence of enteroviral genomes and viral replication in the myocardium, leading to injurious inflammation [31], [32], [33], [34], [35]. In addition, some studies have suggested that chronic heart failure and dilated cardiomyopathy may result from subclinical myocarditis and viral persistence based on the presence of viral genomes in diseased heart specimens (10,11 and reviewed in [36]). Our model provides some support for this hypothesis. The consistent appearance of myocardial pathology in our model makes it useful for investigating these hypotheses and preclinical testing of treatments aimed at preventing the most severe, and often fatal, complications of infection with enteroviruses. This model may also be useful to examine differences in the pathogenicity of different CVB strains. For example, we noted that the CVB3 RNA was detected in the plasma in all three animals infected with CVB3-MCH, compared to only 1 of 3 monkeys infected with a comparable intravenous inoculum of CVB3-H3 (Table 1). By contrast, CVB3_RNA was detected in the myocardium of all three animals infected with CVB3-H3, versus only none of the animals receiving CVB3-MCH. Additional experiments would be needed to confirm these initial results in monkeys, but they are consistent with our having seen a higher morality rate in mice infected by intraperitoneal injection of the MCH strain, compared to the H3 variant [6].

Therapies for enteroviral infection and viral associated cardiomyopathy are limited, and development of these therapeutic approaches has been inhibited by the heterogeneous presentation of enteroviral infection and myocarditis in clinical practice. Murine models are useful, but concerns may be raised about the relevance of murine models to myocarditis in humans. For example, infection of BALB/c mice with myocarditic strains of CVB commonly produces acute myocarditis and dilated cardiomyopathy with lymphocytic infiltration of the myocardium, while myocarditis is far less frequently observed after infection of C57Bl/6 mice [10]. In our view, the non-human primate model we have described represents a distinct advantage over murine models of myocarditis, in which the outcome of infection is substantially influenced by the strain of mouse chosen. The cardiac physiology and of cynomolgous monkeys and non-human primates is also comparable to humans, permitting the use of echocardiography approaches employed in pediatrics. In addition, the immunogenetics of the major histocompatibility loci of cynomolgous monkeys have been well studied, permitting detailed studies of humoral and cellular immune responses [16].

As noted above, no antiviral agents are available for the treatment of severe enteroviral infection, although a trial of pleconaril [37], [38] for the treatment of neonatal disease [Collaborative Antiviral Study Group Protocol 106] was recently completed and is undergoing data analysis. However, a variety of potentially useful compounds have been recently identified [38], including our identification of fluoxetine and other existing medications as inhibitors of CVB replication [39]. The impact of these compounds on disease is so far untested but evaluation in cynomolgous monkeys might prove useful for preclinical evaluation of toxicity and antiviral activity.
